# Strengthening the health workforce and rolling out universal health coverage: the need for policy analysis

**DOI:** 10.3402/gha.v6i0.21852

**Published:** 2013-07-24

**Authors:** Adam D. Koon, Susannah H. Mayhew

**Affiliations:** Department of Global Health and Development, London School of Hygiene and Tropical Medicine, London, UK

**Keywords:** human resources for health, universal health coverage, low- and middle-income countries, policy analysis

## Abstract

This article opens a debate about how to think about moving forward with the emerging twin movements of human resources for health (HRH) and universal health coverage (UHC). There is sufficient evidence to warrant these movements, but actors and the policy process significantly affect which policies are adopted and how they are implemented. How exactly this occurs in low- and middle-income countries (LMICs) is not very well understood. Furthermore, it is not clear whether actors will mobilize for or against the emergent HRH and UHC agendas. Policy analysis should help illuminate potential strategies to account for multiple interests and divergent values in volatile stakeholder environments. We argue that not only should the movement for UHC be paired with current efforts to address the human resources crisis, but also, for both to succeed, we need to know more about how health policy works in LMICs.

The post-2015 global health debate presents an important opportunity to link the move toward strengthening the health workforce in low- and middle-income countries (LMICs) with the march toward universal health coverage (UHC) globally. We feel that this is a timely call as the current crisis in human resources for health (HRH) may be overshadowed and potentially exacerbated by new policies aimed at achieving UHC. What would be the point of rallying behind UHC, for example, if LMIC health systems did not have the staff to ensure its implementation? Also, it is still not clear how certain UHC strategies will affect service delivery. For instance, will new approaches change provider skills, incentives, payment structures, and procedures? Moreover, what impact will these innovations have on quality of care and provider motivation? Any proposal for UHC should be sensitive to the fact that significant investment in strengthening the health workforce, and a proper understanding of the likely workforce consequences of the proposed UHC mechanisms, should be preconditions of UHC's adoption into policy.

Health policy research has shown that actors – and their relative power – play a crucial role in the policy process (1), and the successful implementation of both a health workforce and UHC policy will be greatly influenced by politics. In fact, even international actors and advocacy campaigns, such as the one that is emerging around UHC, can affect health policy at the national level. The process by which this occurs is called ‘policy transfer’ and has been shown to affect country-level policy on tuberculosis (2) and maternal health (3). So, advocacy at the international level matters. Support for UHC at the recent World Health Assembly and the United Nations General Assembly will surely play a role in raising the profile of UHC on the crowded health agenda in many LMICs. Moreover, well-documented experience with installing risk-pooling mechanisms in middle-income countries, such as Mexico and Thailand, may help guide other countries toward UHC.

However, UHC is proving to be a highly politicized pursuit in many countries. This is typical of collective action, where multiple stakeholders cooperate to provide a public good. In this case, access to health services and financial protection from catastrophic health expenditures is the public good, for which a primary instrument may be health insurance. As the design and implementation of the instrument will involve financial and normative decisions, a range of actors will be involved. This requires cooperation between multiple sectors and branches within government, preliminary analyses by technical experts and scientists, and input from civil society about the values that communities feel should be institutionalized in the program design. The media play an important role in shaping public opinion, as do the professionals who will be responsible for carrying out the social mandate. Indeed, policy theories suggest that health providers tasked with implementing policy can shape not only the implementation of policy but ultimately even the policy itself (4).

In parallel to the development of the UHC discourse, a sizable body of evidence is emerging about effective strategies for strengthening the health workforce in LMICs. Newly created health workforce observatories, supported by WHO-AFRO, are beginning to generate reliable metrics about the size and distribution of the health workforce in several African countries (see http://www.hrh-observatory.afro.who.int/). Also, a list of recommendations, based on country experience, for recruiting and retaining health professionals in rural areas is now available (see http://www.who.int/hrh/retention/guidelines/en/index.html). Actors play a significant role in establishing policy to strengthen the size, distribution, and skill mix of the health workforce. Often, multiple actors cooperate to establish medical training centers, coordinate the accreditation of health facilities, and license health professionals. A given arrangement, such as the location of training institutes, quantity of government scholarships, and administrative capacity of regulatory agencies, can have significant ramifications for the size and quality of the health workforce. The distribution of a health workforce requires the input of several complex governing bodies across multiple sectors in the design of compulsory service requirements, incentive packages, and enhanced workplace management strategies. Finally, developing a dynamic health workforce that sensitively addresses the needs of all members of society may call for cadre establishment, task shifting, and supportive supervisory structures to be installed. All of these processes are likely to be heavily contested given the multiplicity of interests, the competing values, and the complexity of the stakeholder environment, which are all characteristic of many LMICs.

Understanding the role and influence of actors in determining both UHC and HRH policies will be critical to achieving the goals of equitable and universal access to services, yet the full range of actors has yet to be explored. In the 1940s, vigorous negotiations between UK Minister of Health Aneurin Bevan and the British Medical Association (5) and US President Harry Truman and the American Medical Association (6) represented potential inflection points in health policy and helped determine the fate of healthcare in their respective countries. Unfortunately, we have little information about how professional associations work in LMICs or how power is negotiated and distributed in the health policy process. Recent physician strikes in India, Ghana, and Mozambique as well as nursing strikes in Kenya have illustrated the important role that professional associations play in collective bargaining. This calls into question the prevailing notion that the health professions are weak and disorganized in LMICs (1, 7). Furthermore, the recent emergence of such entities in LMICs could create difficulties for policy makers who are unaccustomed to a powerful and privileged negotiating block.

The convergence of HRH and UHC has the potential to substantially strengthen LMIC health systems. To achieve this promise, the inclusion of health workforce considerations at the policy development stage is critical. We need a better understanding of the political context and the wide array of actors who will shape policy development and implementation in LMICs as well as of the processes by which decisions are taken and policy coalitions formed. Policy analysis can shed light on these critical issues and help to ensure that the current move toward marrying the urgent HRH and UHC movements can truly strengthen health systems governance, while catalyzing a ‘people-centered’ health policy process.
